# Brain-Wide Mapping of Afferent Inputs to Accumbens Nucleus Core Subdomains and Accumbens Nucleus Subnuclei

**DOI:** 10.3389/fnsys.2020.00015

**Published:** 2020-03-18

**Authors:** Liping Ma, Wenqi Chen, Danfang Yu, Yunyun Han

**Affiliations:** ^1^Department of Neurobiology, School of Basic Medicine and Tongji Medical College, Huazhong University of Science & Technology, Wuhan, China; ^2^Department of Neurology, Provincial Hospital of Integrated Chinese and Western Medicine, Wuhan, China; ^3^Institute for Brain Research, Collaborative Innovation Center for Brain Science, Huazhong University of Science and Technology, Wuhan, China

**Keywords:** nucleus accumbens, brain-wide mapping, afferent input, retrograde tracer, tract-tracing, neuroanatomy

## Abstract

The nucleus accumbens (NAc) is the ventral part of the striatum and the interface between cognition, emotion, and action. It is composed of three major subnuclei: i.e., NAc core (NAcC), lateral shell (NAcLS), and medial shell (NAcMS), which exhibit functional heterogeneity. Thus, determining the synaptic inputs of the subregions of the NAc is important for understanding the circuit mechanisms involved in regulating different functions. Here, we simultaneously labeled subregions of the NAc with cholera toxin subunit B conjugated with multicolor Alexa Fluor, then imaged serial sections of the whole brain with a fully automated slide scanning system. Using the interactive WholeBrain framework, we characterized brain-wide inputs to the NAcC subdomains, including the rostral, caudal, dorsal, and ventral subdomains (i.e., rNAcC, cNAcC, dNAcC, and vNAcC, respectively) and the NAc subnuclei. We found diverse brain regions, distributed from the cerebrum to brain stem, projecting to the NAc. Of the 57 brain regions projecting to the NAcC, the anterior olfactory nucleus (AON) exhibited the greatest inputs. The input neurons of rNAcC and cNAcC are two distinct populations but share similar distribution over the same upstream brain regions, whereas the input neurons of dNAcC and vNAcC exhibit slightly different distributions over the same upstream regions. Of the 55 brain regions projecting to the NAcLS, the piriform area contributed most of the inputs. Of the 72 brain regions projecting to the NAcMS, the lateral septal nucleus contributed most of the inputs. The input neurons of NAcC and NAcLS share similar distributions, whereas the NAcMS exhibited brain-wide distinct distribution. Thus, the NAcC subdomains appeared to share the same upstream brain regions, although with distinct input neuron populations and slight differences in the input proportions, whereas the NAcMS subnuclei received distinct inputs from multiple upstream brain regions. These results lay an anatomical foundation for understanding the different functions of NAcC subdomains and NAc subnuclei.

## Introduction

The nucleus accumbens (NAc) is a basal forebrain structure located ventromedially to the caudoputamen (CP) and ventrolaterally to the septal nuclei (Groenewegen et al., 1999). It is composed of core (NAcC) and shell (NAcS) regions, with the shell regions further subdivided into medial shell (NAcMS) and lateral shell segments (NAcLS; Záborszky et al., 1985; Heimer et al., 1997; Zahm, 1999, 2000; Yang et al., 2018). The NAc is important in many functions (Floresco, 2015), such as learning and memory (Li et al., 2018), reward processing (Carlezon and Thomas, 2009), addiction behavior, locomotor activity, stress-related aversion, liking (Castro et al., 2016), motivation (Castro and Bruchas, 2019), and sexual motivation (Everitt, 1990; Beny-Shefer et al., 2017). In addition, NAc dysfunction is associated with many mental disorders, including schizophrenia (Cotter et al., 2001), Huntington’s disease (Albin et al., 1989), alcohol addiction and drug abuse (Volkow et al., 2007; Lobo et al., 2010; Pirkulashvili et al., 2017; Morales et al., 2019), Alzheimer’s disease (Schliebs and Arendt, 2011; Nie et al., 2017), and depression (Nestler and Carlezon, 2006).

The NAcS also exhibits functional heterogeneity. The NAcMS plays key roles in facilitating the reinforcement of drug abuse, mediating goal-directed behavior, and suppressing unrewarding or irrelevant behaviors (Hoque et al., 2017; Corre et al., 2018), whereas the NAcLS participates in positive motivation (Yang et al., 2018) and reward-directed behavior (Smedley et al., 2019). Furthermore, a functional dissociation exists between the NAcLS and NAcMS in regard to consummatory and motivated behavior (van der Plasse et al., 2012). Although less well characterized, functional heterogeneity also exists within the NAc core (NAcC) subdomains. A previous study has reported that the rostral and caudal NAc responded to serotonin receptor agonists differently (Bowers et al., 2000). Deep brain stimulation of the dorsal NAcC (dNAcC) can facilitate fear extinction, whereas stimulation of the ventral NAcC (vNAcC) below the anterior commissure can enhance fear learning (Rodriguez-Romaguera et al., 2012). These results are difficult to interpret, however, as later reports have indicated that the same stimulation parameters in the dNAcC can enhance drug-seeking (Martínez-Rivera et al., 2016). Investigations on connectivity patterns, including input and output circuits, can help dissect the diverse functions of the NAc subregions. The output patterns of the NAc have been well identified, including both the direct and indirect pathways (Kupchik et al., 2015; Gould et al., 2017). Furthermore, previous studies on afferent connections to the NAc subregions have primarily focused on the hippocampus, basal amygdala, and ventral mesencephalon (Zahm and Brog, 1992). However, the detailed organization patterns of upstream circuits across the brain, especially direct inputs to the subregions of the NAcC and NAcS remain unclear.

Here, we retrogradely labeled subregions of the NAcC and NAcS with cholera toxin subunit B conjugated with Alexa Fluor. Using a fully automated slice scanning system and interactive framework for brain-wide mapping (i.e., WholeBrain; Fürth et al., 2018), we systematically characterized the brain-wide inputs to the NAc subregions, including the rostral, caudal, dorsal, and ventral subdomains of NAcC (rNAcC, cNAcC, dNAcC, and vNAcC) and NAcMS and NAcLS.

## Materials and Methods

### Animals

Twenty 6-week-old wild type C57BL/6J mice were purchased from the Beijing Vital River Laboratory Animal Technology Company Limited (China). The animals were housed 3–5 mice/cage (30 cm × 18 cm × 13 cm) under a 12 h:12 h light-dark cycle (light on at 8:00 am), with *ad libitum* access to rodent food and water in an environmentally controlled room at a consistent ambient temperature (23 ± 2°C) and humidity (50% ± 5%). The mice used in the study were adult (8–10 weeks) male mice.

### Ethics Approval

This study was carried out in accordance with the guidelines issued by the Institutional Animal Care and Use Committee (IACUC) at Huazhong University of Science and Technology, Wuhan, China. All protocols were approved by the IACUC and every effort was made to ensure the mice used were treated humanely and any discomfort was kept to a minimum.

### Microinjection and Stereotactic Surgery

CTB-conjugated Alexa Fluor 488 (CTB-488) and Alexa Fluor 555 (CTB-555) were purchased from Thermo Fisher Scientific, Waltham, MA, USA. The tracer was dissolved in neutral phosphate-buffered saline (PBS) at a concentration of 1 μg/μl, aliquoted at 5 μl each and stored at −20°C until usage.

Dexamethasone (30 nl, 2 mg/ml, intraperitoneal injection) was given to the mice half an hour before surgery. Then, they were anesthetized with 5% chloral hydrate (0.1 ml/10 g) before the CTB injection, with a simultaneous intraperitoneal injection of 30 μl of atropine (0.1 μg/μl) and scalp infiltration anesthesia of lidocaine at a concentration of 5 μg/ml. Supplementary doses of chloral hydrate were given throughout the procedure as needed. After the mice were completely anesthetized, they were fixed on a stereotactic stent (68030, RWD Life Science, China) and kept warm (37°C) with an electric heating pad (RWD Life Science, China). Before adjusting their skulls in parallel to the reference panel, their eyes were covered with eye lube. A 0.5-mm diameter drill bit was used to make a small hole in the skull above the target area. To label upstream inputs to the NAcC, CTB-488 and CTB-555 were stereotactically injected into the right rNAcC (coordinates: AP: +1.8 mm, ML: −1.1 mm, DV: −3.75 ± 0.15 mm) and cNAcC (coordinates: AP: +0.9 mm, ML: −1 mm, DV: −3.9 ± 0.15 mm), respectively, using a glass pipette connected to a pneumatic pump (PV820, pneumatic pico-pump, World Precision Instruments Inc., Sarasota, FL, USA). To label upstream input to the NAcS, CTB-488 and CTB-555 were stereotactically injected into the right NAcMS (coordinates: AP: +1.3 mm, ML: −0.55 mm, DV: −4.2 mm) and NAcLS (coordinates: AP: +1.3 mm, ML: −1.7 mm, DV: −4.15 mm), respectively. 30 nl of CTB solution was slowly injected (6 nl/min) into each injection site with an impulse injection (20 psi at 5–10 Hz with a pulse duration of 10–15 ms). A low positive “holding” pressure was maintained in the injecting pipette between injection pulses to prevent fluid up­take through capillary action. After the last pulse was given, the glass electrode was held at the injection site for 10 min and then slowly retracted. After the injection, the surgical site was rinsed with saline, sutured and disinfected with iodophor. The operated mice were then placed on a heating pad until fully awake. The mice were given 0.03 ml of ketorolac tromethamine analgesic (1 μg/μl) and 0.03 ml of anti-inflammatory drug enrofloxacin (0.5%, Baytril, Bayer Bitterfeld GmbH, Germany) daily in the next 3 days.

### Tissue Processing

Two weeks after CTB injection, the mice were deeply anesthetized by an intraperitoneal overdose injection of chloral hydrate, followed by transcardial perfusion with 100 ml of 0.1 M PB and 4% paraformaldehyde (PFA) in 0.1 M phosphate buffer (PB). Mouse brains were carefully removed and then post-fixed with 4% PFA in 0.1 M PB overnight at 4°C. The brains were placed in a 20% sucrose-0.1 M PB solution at 4°C until they sank, then moved to a 30% sucrose-0.1 M PB solution at 4°C until they sank. The brains were sectioned coronally (30-μm thickness) with a freezing microtome (Leica Microsystems, Wetzlar, Germany). One out of every four sections was collected and kept in 0.01 M PBS in a 48-well plate and then mounted on a glass slide. These sections were imaged for all subsequent analyses with a fully automated slice scanning microscope (10× objective, NA 0.4, Olympus VS120, Japan) at a resolution of 0.67 μm. All images were saved as 16 bit grayscale in non-compressed “.tif” format.

### Cell Counting and Input Brain Region Identification

We used 30-μm sections from 10 brains to perform cell counting with ImageJ software. For cell counting in each area, we loaded the image into ImageJ and used its Cell Counter multi-point tool to mark the soma. We counted all long-range upstream brain regions in the ipsilateral hemisphere of the injection sites. The pixel position of each marked cell in each brain section was exported as a .csv file.

The interactive WholeBrain^1^ framework is an R-language based open-source software developed by Fürth et al. (2018). We transform the .csv raw data into R data for further analysis in the interactive WholeBrain framework. After images with cell counting information were loaded into the interactive WholeBrain framework, it automatically loaded the corresponding Allen Brain Atlas for registration. Mostly, after auto-registration, the actual image and Atlas did not match well (Figure 2Bi). However, the framework provides an interface to allow manual adjustment of the atlas to match the image by overlaying their landmarks lateral ventricle (VL), anterior commissure, anterior part (aca) to the image (Figure 2Bii).

**Figure 1:**
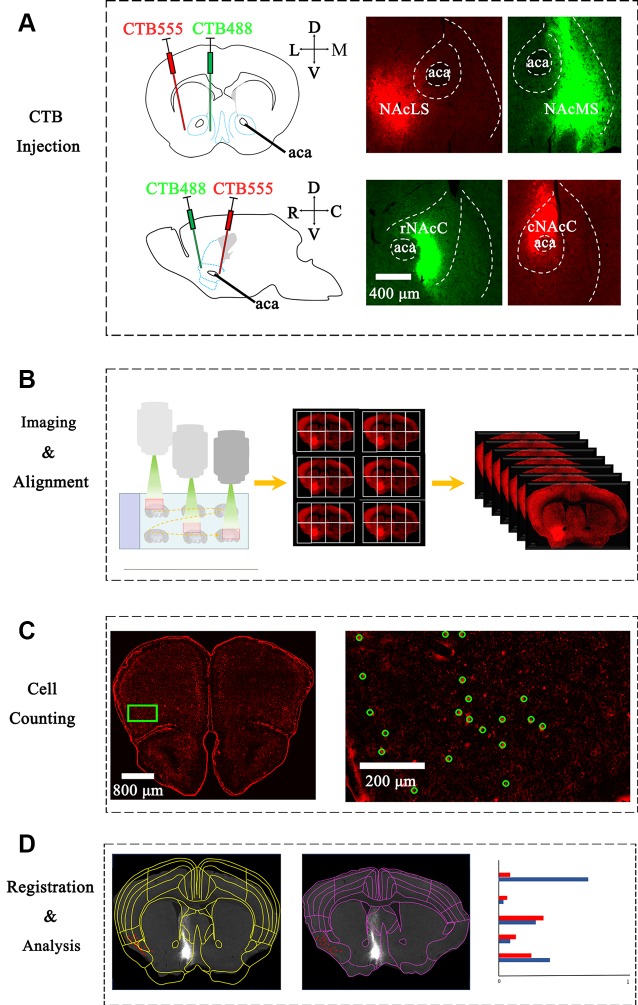
Experimental strategy for identification of inputs to different nucleus accumbens (NAc) subregions. **(A)** Upper panel: left, schematic injection site in NAcS; middle, representative coronal brain sections near CTB-conjugated Alexa Fluor 555 (CTB-555) (red) injection sites in lateral shell segments (NAcLS); Right, representative coronal brain sections near CTB-488 (green) injection sites in medial shell segments (NAcMS). Lower panel: Left, schematic injection site in NAc core (NAcC); middle, representative coronal brain sections near CTB-488 (green) injection sites in rNAcC; right, representative coronal brain sections near CTB-555 (red) injection sites in Caudal nucleus accumbens core (cNAcC). **(B)** The scheme of imaging serial sections by an automated slice scanning microscope. Manual alignment of whole-brain serial images along the rostral-caudal axis. **(C)** Manual counting of CTB-labeled neurons of each slice using ImageJ. Green circles marked neurons labeled by CTB-555. **(D)** Left, registration of Allen Atlas regions to one section; middle, manual adjustment to fit atlas segmentation to the image. Note that the adjusted segmentation resulted in the different categorization of individual cell bodies (red dot). Right: schematic of statistical analysis of WholeBrain data.

**Figure 2:**
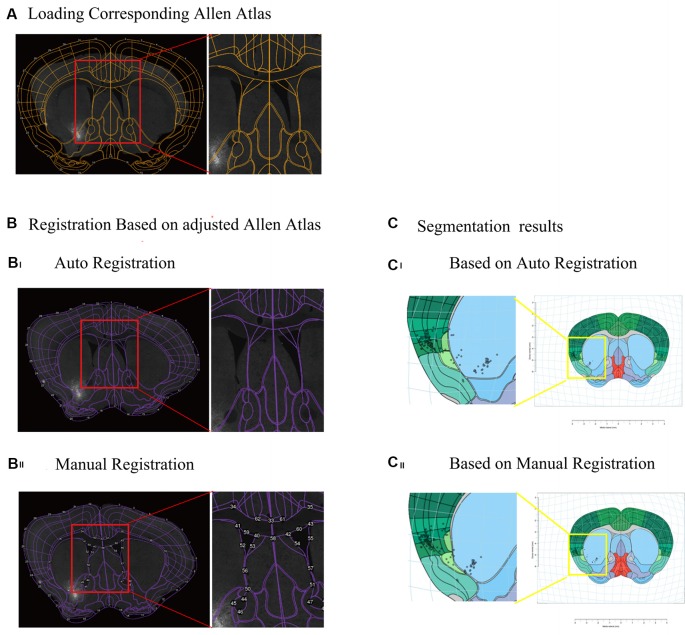
Registration of atlas and segmentation of brain region using interactive WholeBrain framework. **(A)** Loading the image of one brain section and corresponding Allen Atlas in the WholeBrain framework. Orange outlines indicate borders of the ventricles in the atlas (see right insert), which do not match the corresponding image underneath it. **(B)** Registration of Allen Atlas. Numbered points on purple lines indicate adjustable anchor points of Allen Atlas. **(Bi)** Auto registration of Allen Atlas to the image in the WholeBrain framework. The outline of atlas well matches the outline of the section, but the border of the lateral ventricle (VL) does not match the location of the VL of the section (see right insert). **(Bii)** Manual registration of Allen Atlas to the section. After manually adjusting borders to match the locations of landmarks in the section, e.g., lateral VL, anterior cingulate area (ACA), the atlas well matched the section, including lines indicating lateral ventricles (see right insert). **(C)** Comparison of segmentation results of registration, showing that labeled cells are assigned to different brain regions (see left insert). **(Ci)** Auto registration. **(Cii)** Manual registration.

Only the regions containing a significant number of labeled cells (i.e., more than 10) were considered as input regions for further analysis (Luo et al., 2019). The input from each upstream region was normalized by dividing the number of labeled neurons found in that region by the total number of labeled neurons from each injection site in each brain, which was then called the proportion of total inputs. When performing a correlation analysis between the co-labeled neurons and the total labeled neurons projecting to the NAcC subdomains and the NAcS subnuclei, proportions of co-labeled neurons were calculated by dividing the number of co-labeled neurons found in that major area by the total labeled neurons from one brain. The total labeled neurons of one major area came from CTB-488 labeled neurons plus CTB-555 labeled neurons then minus the co-labeled neurons in that area.

### Statistical Analysis

All values were presented as Mean ± SEM, with *P < 0.05, **P < 0.01, ***P < 0.001, ****P < 0.0001. Unpaired two-tailed Student’s *t-test* was performed when comparing inputs between the two groups. One-way analysis of variance (ANOVA) with Dunnett’s *post hoc* test for single factors was performed when comparing inputs among three or more groups, whereas two-way ANOVA followed by multiple comparisons with Dunnett’s *post hoc* test was used for double factor experiments. To quantify the similarity in input patterns, we calculated Pearson’s correlation coefficients.

## Results

### Tracing Whole-Brain Inputs to NAcC Subdomains and NAcS Subnuclei

We stereotaxically injected the retrograde tracer CTB into the NAc subnuclei to map the brain-wide distribution patterns of input neurons. Both CTB-488 and CTB-555 (30 nl/injection site) were stereotaxically microinjected into the rNAcC and cNAcC or NAcLS and NAcMS, respectively (Figure 1A and Supplementary Figures S1, S2). We injected at two depths to label NAcC input neurons, simultaneously allowing us to analyze the input neurons of the vNAcC and dNAcC too. CTB was taken up by the axonal terminals at the injection site and then retrogradely transported to the somata. The neuronal somata across the brain projecting to the rNAcC were labeled with CTB-488 (green), whereas the cNAcC-projecting neurons were labeled with CTB-555 (red). Two weeks after injection, the mice were transcardially perfused, and their brains were fixed and coronally sectioned at a thickness of 30 μm. The CTB-labeled neurons were concentrated in the ipsilateral site, with sparsely labeled neurons also observed in the contralateral hemisphere (data not shown).

To generate overall brain-wide distribution of the CTB-labeled somata, we imaged every fourth brain section with an automated slice scanning system (Figure 1B). The brain slice images were manually aligned along the rostral-caudal axis (Figure 1B). The CTB-labeled neurons of each slice were manually marked using ImageJ, with the results exported and converted to R format for subsequent analysis (Figure 1C). The Allen Brain Atlas at the corresponding rostral-caudal position was registered to each image in the aligned brain-wide stack using the interactive WholeBrain framework (Fürth et al., 2018; Figures 1D, 2). This framework allowed manual tweaking of the atlas to match the images according to the cytoarchitectural landmarks in the brain (Figure 2B). The improvement in cell body segmentation after manual correction is shown in Figures 1D, 2C.

**Figure 3:**
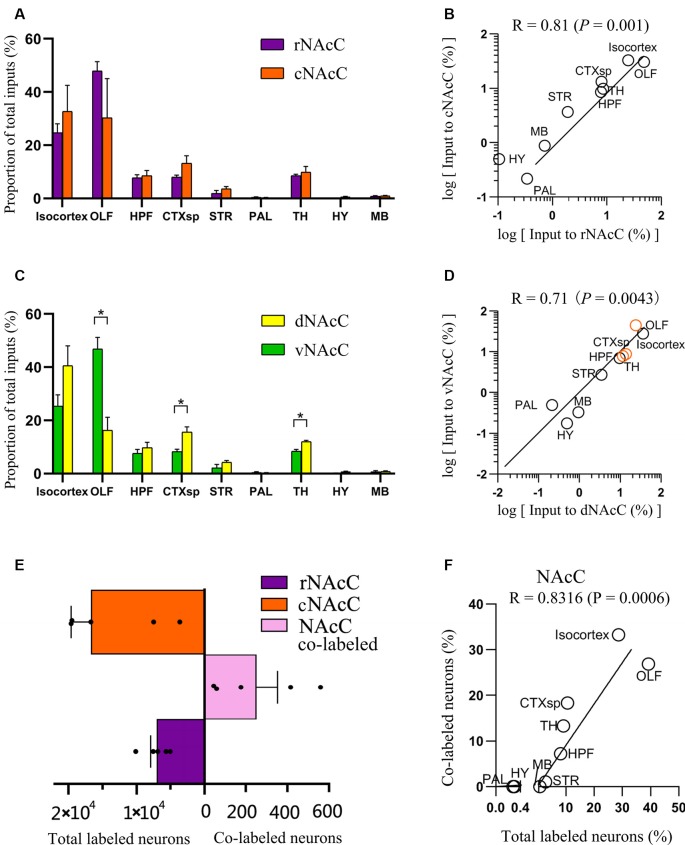
Overview of whole-brain inputs to subdomains of NAcC. **(A)** Distribution of input neurons of rNAcC and cNAcC across nine major brain areas [two-way analysis of variance (ANOVA), *n* = 5 mice for rNAcC and cNAcC]. **(B)** Correlation of the distributions of inputs neuron of rNAcC and cNAcC across nine major brain areas. **(C)** Distribution of input neurons of dNAcC and vNAcC across nine major brain areas (two-way ANOVA, *n* = 4 mice for dNAcC and vNAcC). **(D)** Correlation of the distributions of inputs neuron of dNAcC and vNAcC across nine major brain areas. The brain areas containing significantly different proportions of the input neurons projecting to two NAcC subdomains were highlighted as orange circles. **(E)** The number of total and co-labeled input neurons of rNAcC and cNAcC. **(F)** Correlation of fraction of co-labeled input neuron in each area and the proportion of input neurons contributed by such brain area to the total inputs to NAcC. *P < 0.05.

We calculated the number of CTB-labeled neurons in each brain region. Brain regions with more than 10 labeled cells, which equated to 0.1% of all labeled neurons across the brain, were included for quantitative analysis. The median number of whole-brain labeled neurons to the NAc subnuclei was 9,518 (6,878, 7,566, 5,057, 5,654, and 10,121 to the rNAcC; 7,493, 3,661, 16,728, 19,508, and 19,617 to the cNAcC; 10,988, 11,275, 9,450, 7,192, and 10,383 to the NAcLS; and, 9,585, 9,264, 8,439, 14,997, and 17,530 to the NAcMS). The median number of co-labeled neurons to the NAcC subdomains was 175 (558, 175, 58, 44 and 414), and the median number of co-labeled neurons to the NAcS was 11 (11, 14, 6, 17 and 0; Figures 3E, 6E). To minimize the influence of experimental variation on the total number of labeled neurons, the input from each region was normalized by dividing the number of labeled neurons found in that region by the total number of labeled neurons in each injection site to obtain the proportion of total inputs. In total, 75 input regions were compared. Among them, 57 brain regions projecting to the NAcC, 55 to the NAcLS, and 72 to the NAcMS. The 75 brain regions could be grouped into nine major brain areas, including the isocortex, olfactory areas (OLF), hippocampal formation (HPF), cortical subplate (CTXsp), striatum (STR), pallidum (PAL), thalamus (TH), hypothalamus (HY), and midbrain (MB). Thus, these results indicate that the NAc (including the NAcC and NAcS) neurons integrated inputs from diverse brain regions, ranging from the cerebrum to the brain stem.

**Figure 4:**
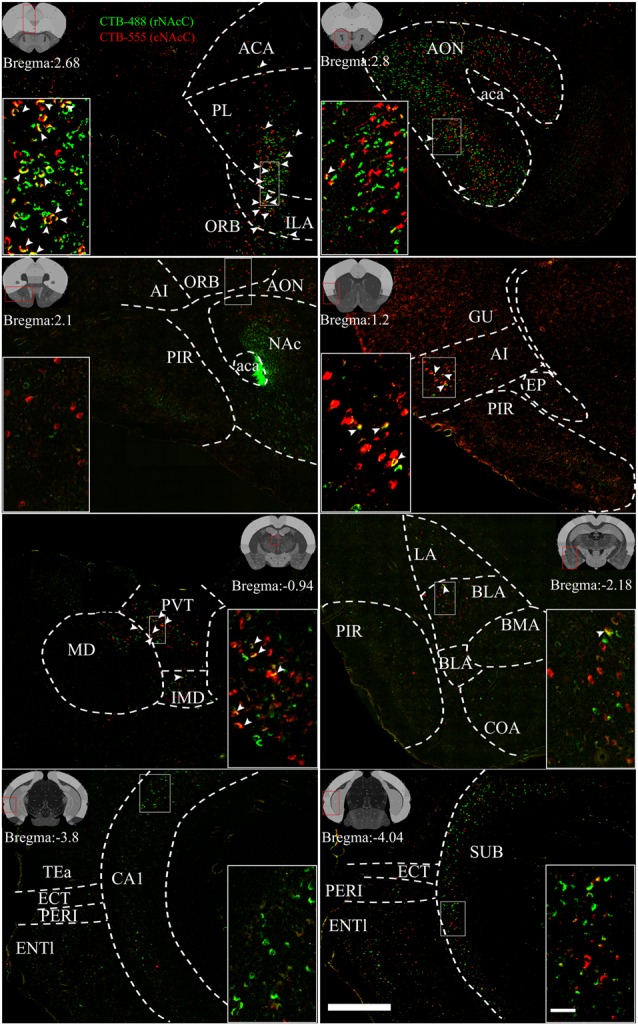
Reprehensive images of input neurons of NAcC subdomains. Representative coronal sections showing CTB-labeled inputs to rNAcC (green) and cNAcC (red). The region in the white box is shown at a higher resolution in the inset. The location of each section is indicated by the red box in the atlas template image (gray) in the upper left or upper right corner. Scale bar = 400 μm, 50 μm (inset).

**Figure 5:**
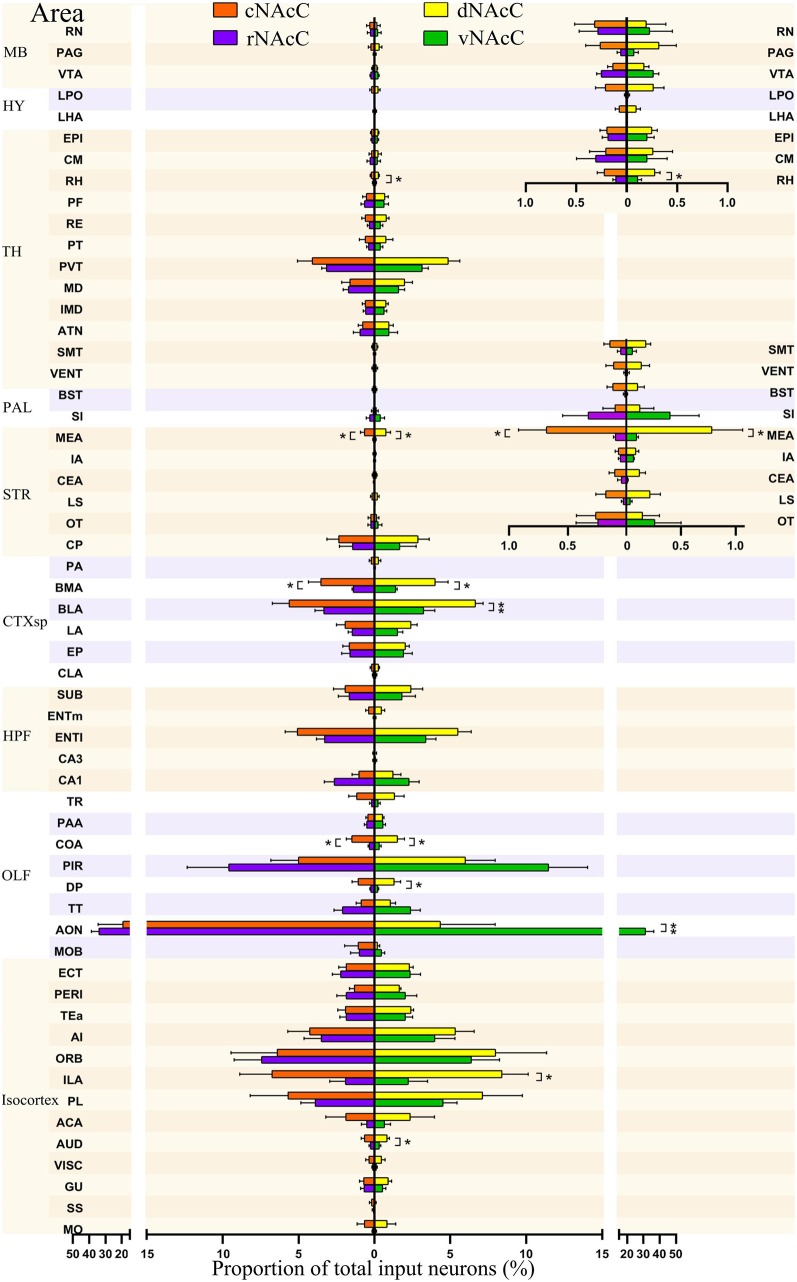
Input neurons of NAcC subdomains share similar distribution patterns across the brain. The proportions of total inputs contributed by each brain area to each NAcC subnuclei, including rNAcC (purple), cNAcC (red), dNAcC (yellow) and vNAcC (green). The proportions of input neurons in midbrain (MB), thalamus (TH), and hypothalamus (HY; superior) and TH, pallidum (PAL), and striatum nuclei (inferior) are shown with finer-scale as an inset on the upper right corner (Student’s *t*-test, *n* = 5 mice for rNAcC and cNAcC, *n* = 4 mice for dNAcC and vNAcC). *P < 0.05, **P < 0.01.

**Figure 6:**
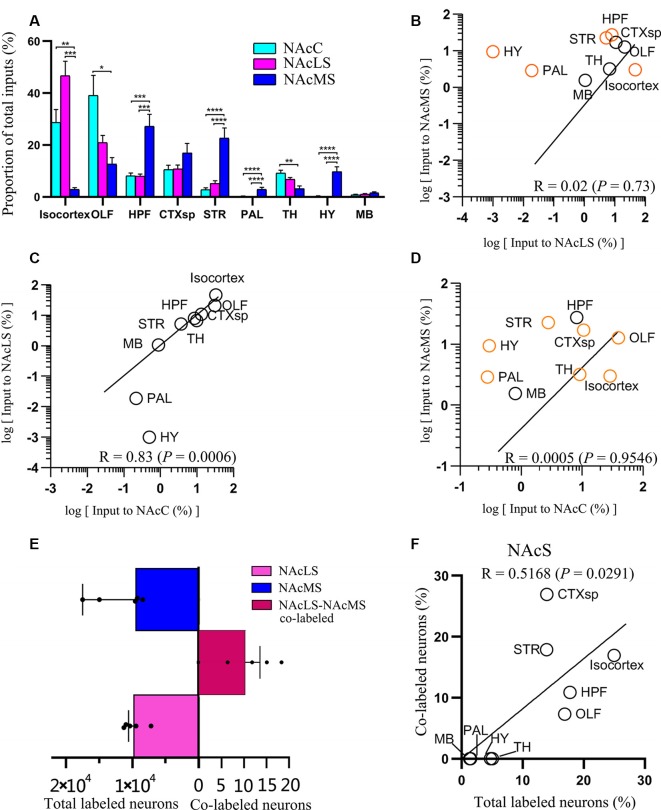
Overview of whole-brain inputs to NAcMS, NAcLS, and NAcC. **(A)** Distribution of input neurons of NAcC, NAcLS, and NAcMS across nine major brain areas (two-way ANOVA, *n* = 5 mice each). **(B–D)** Correlation of the distributions of inputs neurons of NAcLS, NAcMS, and NAcC. The brain areas with significantly different contributions to the input neuron of two subnuclei were highlighted by orange circles. **(E)** The number of total and co-labeled input neurons of NAcLS and NAcMS. **(F)** Correlation of fraction of co-labeled input neuron in each area and the proportion of input neurons contributed by such brain area to the total inputs to NAcS. *P < 0.05, **P < 0.01, ***P < 0.001, ****P < 0.0001.

### Global Distributions of Input Neurons to NAcC Subdomains (rNAcC vs. cNAcC and dNAcC vs. vNAcC) Are Similar

The distributions of input neurons projecting to the rNAcC and cNAcC across the nine major brain areas were similar (two-way ANOVA; Brain areas × Subdomain_r-c_, *F*_(8,72)_ = 1.30, *P* = 0.26; Brain areas, *F*_(8,72)_ = 19.34, *P* < 0.0001; Subdomain_ r-c_, *F*_(1,72)_ = 5.02 × 10^−6^, *P* > 0.99; Figure 3A), whereas the distributions of neurons projecting to the dNAcC and vNAcC were slightly different (two-way ANOVA; Brain areas × Subdomain_d-v_, *F*_(8,54)_ = 10.61, *P* < 0.0001; Brain areas, *F*_(8,54)_ = 44.54, *P* < 0.0001; Subdomain_d-v_, *F*_(1,54)_ = 1.67 × 10–^6^, *P* > 0.99; Figure 3C). We also found a larger proportion of OLF neurons projecting to the vNAcC (46.93% ± 4.29%) than to the dNAcC (16.30% ± 4.89%), whereas the neurons projecting from the CTXsp and TH to the dNAcC (15.56% ± 2.03% and 12.02% ± 0.46%, respectively) were more than that to the vNAcC (8.25% ± 0.94% and 8.44% ± 0.43%, respectively). We analyzed the correlation of inputs distributions to the NAcC subdomains (i.e., rNAcC vs. cNAcC and dNAcC vs. vNAcC). The squared Pearson’s correlation coefficient (R) for inputs between rNAcC and cNAcC was 0.81 (*P* = 0.001; Figure 3B), and that between dNAcC and vNAcC was 0.71 (*P* = 0.0043; Figure 3D, the source regions with differential input portions are highlighted in orange circles).

rNAcC and cNAcC shared similar input patterns but very few co-projecting input neurons. Figure 4 shows representative coronal images of the CTB retrogradely labeled neurons in the upstream brain regions. The CTB-488- and CTB-555-labeled neurons indicated populations projecting to the rNAcC and cNAcC, respectively. Notably, in the same brain region, most neurons that projecting to the rNAcC were not the same population that projecting to the cNAcC. Of all neurons projecting to the rNAcC and cNAcC, the proportion of co-labeled neurons was only 1.49% ± 0.70%. Specifically, correlation analysis showed that the proportions of total labeled neurons and the proportions of co-labeled neurons projecting to the NAcC of corresponding brain regions were closely related (*R* = 0.8316, *P* = 0.0006; Figure 3F). Thus, the proportions of co-labeled neurons were not brain-region selective but appeared to be related to total inputs to the NAcC. These results suggest that the rNAcC and cNAcC share common upstream regions but receive input from relatively distinct neuronal populations of each upstream brain region.

### Comparison of Inputs to rNAcC vs. cNAcC and vNAcC vs. dNAcC Among 57 Upstream Brain Regions

We further divided the nine major brain areas into finer segmented brain regions and found that input neurons of the NAcC were observed in 57 of them. The distribution of input neurons projecting to the rNAcC differed (*P* < 0.0001, *F*_(56,228)_ = 30.95, one-way ANOVA; Figure 5, left), with the anterior olfactory nucleus (AON) contributing most of the inputs (34.05% ± 4.68%), followed by the piriform area (PIR, 9.59% ± 2.74%) and orbital area (ORB, 7.45% ± 1.79%). The neurons projecting to the cNAcC also differed (*P* = 0.0007, *F*_(56,228)_ = 1.876, one-way ANOVA), with the AON contributing most of the inputs (19.31% ± 15.23%), followed by the infralimbic area (ILA, 6.76% ± 2.12%) and ORB (6.42% ± 3.03%). Most regions showed no statistical differences in their contribution to input neurons projecting to the rNAcC and cNAcC (Student’s *t*-test), except the medial amygdalar nucleus (MEA, *P* = 0.04), basomedial amygdalar nucleus (BMA, *P* = 0.03), and cortical amygdalar area (COA; *P* = 0.01).

Among the 57 upstream regions projecting to the NAcC, the distribution of input neurons projecting to the dNAcC differed (*P* < 0.0001, *F*_(56,171)_ = 5.74, one-way ANOVA; Figure 5, right), with the ILA contributing most of the inputs (8.41% ± 1.71%), followed by the ORB (7.97% ± 3.36%) and prelimbic area (PL, 7.13% ± 2.61%). The distribution of input neurons projecting to the vNAcC also differed (*P* < 0.0001, *F*_(56,171)_ = 24.61, one-way ANOVA), with the AON contributing most of the input source (31.38% ± 4.97%), followed by the PIR (11.48% ± 2.56%) and ORB (6.38% ± 1.86%). Comparing the upstream regions to the dNAcC and vNAcC, only 9 out of 57 regions showed statistical differences in the input proportion (Student’s *t*-test), including the auditory area (AUD; *P* = 0.03), ILA (*P* = 0.03), AON (*P* = 0.0045), dorsal peduncular area (DP, *P* = 0.04), COA (*P* = 0.04), basolateral amygdalar nucleus (BLA, *P* = 0.009), BMA (*P* = 0.02), MEA (*P* = 0.049), and rhomboid nucleus (RH, *P* = 0.004). Overall, the distribution of input neurons to the NAcC subdomains was very similar; therefore, we considered the NAcC, as a whole, to compare to NAcS subnuclei.

### Global Distribution of Input Neurons to NAcLS Is Similar to NAcC But Different From NAcMS

Neurons projecting to the NAcC, NAcLS, and NAcMS exhibited distinct distributions across the nine major brain areas (two-way ANOVA; Brain areas × Subnuclei_C-L-M_, *F*_(16,153)_ = 10.24, *P* < 0.0001; Brain areas, *F*_(8,153)_ = 23.41, *P* < 0.0001; Subnuclei_C-L-M_, *F*_(2,153)_ = 6.44 × 10–^4^, *P* > 0.99). Among them, the OLF contributed most of the afferent inputs (39.18% ± 7.66%) to the NAcC, followed by the isocortex (28.67% ± 5.06%) and CTXsp (10.56% ± 1.65%; Figure 6A). For the NAcLS, the isocortex contributed most of the afferent inputs (46.69% ± 5.58%), followed by the OLF (20.90% ± 2.80%) and CTXsp (10.75% ± 1.52%). For the NAcMS, the HPF contributed most of the inputs (27.22% ± 4.63%), followed by the STR (22.63% ± 3.92%) and CTXsp (16.95% ± 3.70%). We found that the NAcC and NAcLS both received more inputs from the isocortex and OLF, whereas the NAcMS received more inputs from the HPF, CTXsp, and STR. Both the PAL and HY sent preferential innervation to the NAcMS (2.88% ± 0.82%, 9.43% ± 1.92%, respectively).

We quantified the correlations among inputs to the NAcC and NAcS.We pair-wise compared inputs to the NAcC, NAcLS, and NAcMS in the nine major brain areas, with each circle in the scatter plot representing one input brain area (Figures 6B–D). The squared Pearson’s correlation coefficient (R) for inputs between NAcLS and NAcMS was 0.02 (*P* = 0.73; Figure 6B), between NAcC and NAcLS was 0.83 (*P* = 0.0006; Figure 6C), and between NAcC and NAcMS was 0.0005 (*P* = 0.9546; Figure 6D), indicating that the NAcLS shared similar distributions of input neurons to the NAcC but distinct from the NAcMS.

### NAcLS and NAcMS Shared Different Input Patterns Nor Very Few Co-projecting Input Neurons

**Figure 7:**
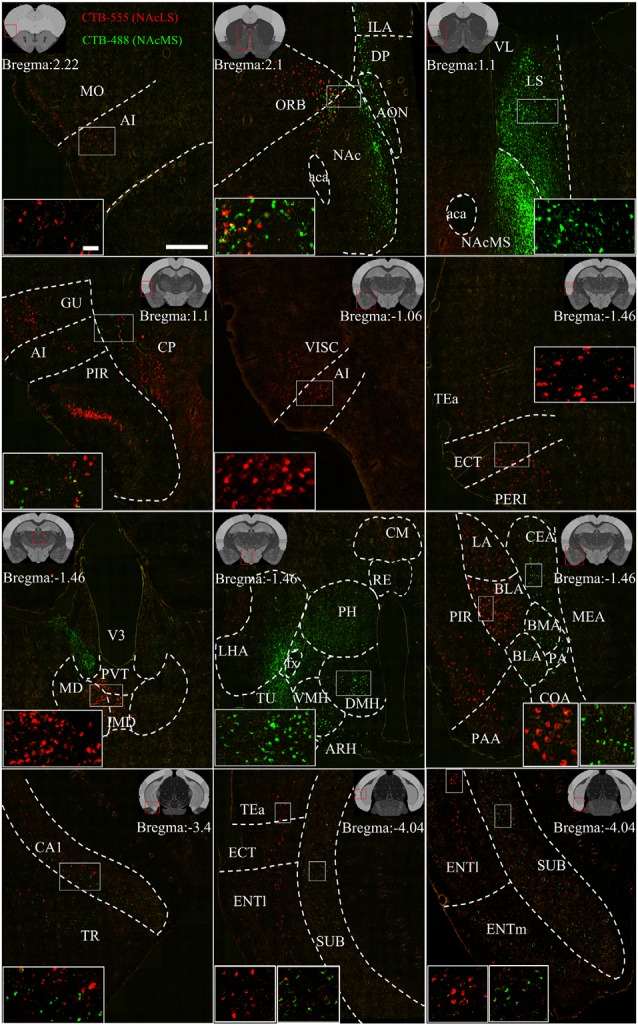
Representative images of input neurons to NAcS subnuclei. Representative coronal sections showing CTB-labeled inputs to NAcLS (red) and NAcMS (green). The region in the white box is shown at a higher resolution in the inset. The location of each section is indicated by the red box in the atlas template image (gray) in the upper left or upper right corner. Scale bar = 400 μm, 50 μm (inset).

Figure 7 shows representative coronal images of retrogradely labeled input neurons projecting to the NAcLS and NAcMS. The upstream brain regions projecting to the NAcLS and NAcMS were partially different. Notably, some regions only projecting to one of the two areas. A very small number of neurons were found to project to both NAcS subnuclei, accounting for 0.05% ± 0.01% of total input neurons to the NAcS. Correlation analysis showed that the proportions of total labeled neuron and the proportions of co-labeled neurons projecting to the NAcS of corresponding input regions were closely related (*R* = 0.5168, *P* = 0.0291; Figure 6F), indicating that the amount of co-labeled neurons did not depend on the input region but proportion to the total inputs to the NAcS.

### Comparison of Inputs to NAcC, NAcLS, and NAcMS Among 75 Upstream Brain Regions

**Figure 8:**
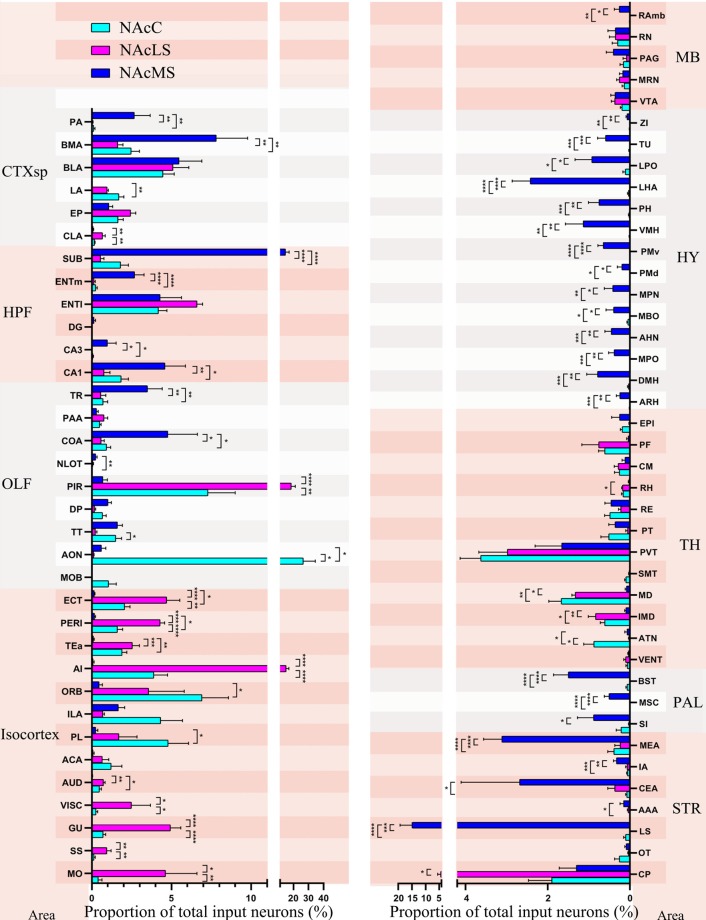
Input distribution of NAcMS differs from NAcLS and NAcC. The proportions of total inputs contributed by each brain area to NAcC, NAcLS, and NAcMS (one-way ANOVA, *n* = 5 mice each). *P < 0.05, **P < 0.01, ***P < 0.001, ****P < 0.0001.

We found in total 75 regions projecting to the NAcC, NAcLS, and NAcMS (57, 55 and 72 brain regions respectively). The distribution of input neurons across those brain regions to NAcC, NAcLS and NAcMS differed from each other (one-way ANOVA; *P* < 0.0001 *F*_(56,513)_ = 10.54, *P* < 0.0001 *F*_(74,300)_ = 24.82 and *P* < 0.0001 *F*_(74,300)_ = 12.11, respectively; Figure 8). The AON contributed the most input neurons projecting to NAcC (26.68% ± 7.90%), followed by the PIR (7.31% ± 1.73%) and ORB (6.94% ± 1.67%). The PIR contributed most of the inputs (18.55% ± 2.64%) to NAcLS, followed by the agranular insular area (AI, 15.11% ± 1.81%) and lateral entorhinal area (ENTl, 6.60% ± 0.35%). The lateral septal nucleus (LS) contributed most of the inputs (14.97% ± 4.37%) to NAcMS, followed by the subiculum (SUB, 14.79% ± 2.37%), BMA (7.80% ± 1.98%) and BLA (5.48% ± 1.43%). 53 out of 75 upstream brain regions contributed different proportions of input neurons to the NAcC, NAcMS, and NAcLS (one-way ANOVA, Supplementary Tables S1, S2). In each major area, the preference for innervating NAc subnuclei was different. For example, the isocortex is preferentially sent axons to the NAcLS, with very little innervation to the NAcMS, except the medial prefrontal cortex (mPFC, including the anterior cingulate area (ACA), PL, ILA, and ORB). The OLF provided almost over 20% of the input neurons to NAcLS and NAcC but only 10% of those to the NAcMS (Figure 6A). Notably, the distribution of input neurons within OLF was not even, with AON and PIR took the most portion of inputs to NAcC and NAcLS (26.68% ± 7.90% and 18.55% ± 2.64% respectively, Figure 8). The HPF and STR provided the largest portions of inputs neurons to NAcMS but only a modest contribution to NAcLS and NAcC (Figure 6A). The SUB, CA1and ENTm (medial entorhinal area) in the HPF provided much more inputs to NAcM than to NAcLS and NAcC, with the SUB took the largest portion all over the brain (14.79% ± 2.37%). The LS, CEA, and MEA in the STR contained much larger portions of NAcMS projecting neurons (14.97% ± 4.37%, 2.68% ± 1.43% and 3.11% ± 0.45%, respectively), whereas the CP contributed more inputs to NAcLS. All regions in the HY and PAL showed preferential innervation to the NAcMS, especially the HY, in which all regions demonstrated exclusive innervation to the NAcMS. All regions in the TH showed similar distribution patterns of input neurons projecting to the NAcMS, NAcLS and NAcC, with each region containing a relatively smaller portion of neurons projecting to NAcMS, as confirmed by correlation analysis (NAcMS vs. NAcLS, *R* = 0.64, *P* = 0.0018; NAcMS vs. NAcC, *R* = 0.73, *P* = 0.0004; NacC vs. NacLS, *R* = 0.90, *P* < 0.0001). For the MB, each region in this area contained a similarly small proportion of projecting neurons to each NAc subnucleus.

### Summary of Distribution of Input Neurons to Subregions of NAcC and NAcS

**Figure 9:**
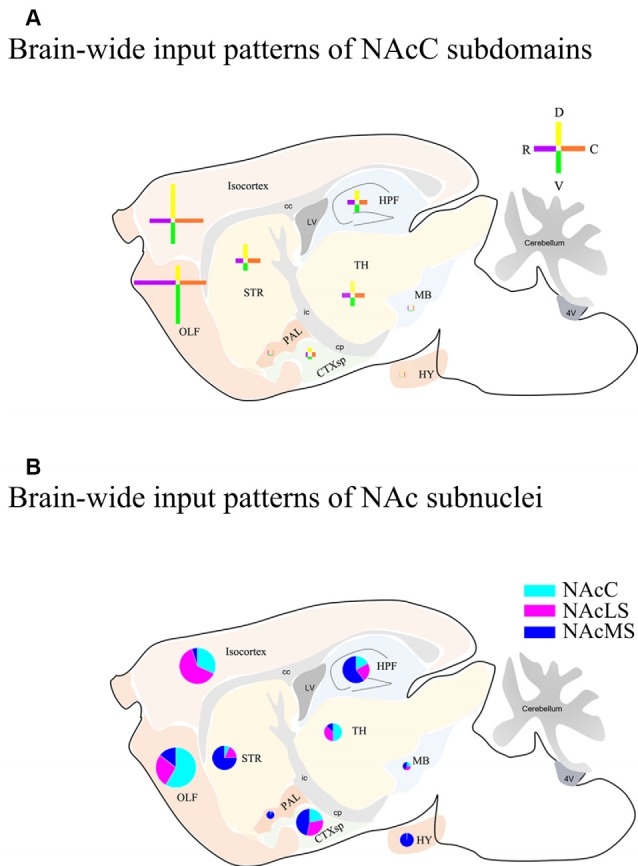
Schemes of brain-wide input patterns of NAcC and NAcS subdomain. **(A)** Brain-wide input patterns of NAcC subdomains. Each cross indicates inputs to NAcC subdomains from corresponding major brain areas and the arm length indicates the relative amount of input neurons in the corresponding major brain area. The horizontal arm shows the relative input amount of rNAcC (purple) and cNAcC (orange), and the vertical arm shows the relative amount of input neurons of dNAcC (yellow) and vNAcC (green). **(B)** Brain-wide input patterns of NAcLS, NAcMS, and NAcC. Every pie indicates the relative amount of input neurons of NAc subnuclei from the corresponding major brain area.

Overall, we compared the brain-wide input patterns of the NAcC subdomains (Figure 9A), most of which received inputs from different neuronal populations in the same upstream brain regions and with a little difference in the proportion of projecting. Comparing the brain-wide input patterns of the NAcC and NAcS subnuclei (Figure 9B), we found that: (1) the brain-wide input patterns of the NAcC and NAcLS were similar, with the main difference being the proportion of input neurons from the same upstream brain region. As shown in Figure 8, most upstream regions projecting to the NAcLS also contain neurons sending inputs to the NAcC, and most often, in the same upstream brain region, the proportion projecting to the NAcLS was greater than that to the NAcC, except for the AON, TT, and ATN; and (2) the NAcMS had a distinct distribution of upstream neurons across the brain compared with the NAcC and NAcLS. In the cerebrum, the isocortex and OLF neurons preferred to send innervation to the NAcLS and NAcC, whereas the HPF, CTXsp, STR, and PAL contained more neurons projecting to the NAcMS. The brain stem TH demonstrated preferential innervation to the NAcC and NAcLS, whereas the HY showed preferential innervation to the NAcMS.

## Discussion

We mapped the organization of input neurons projecting to different NAcC (rNAcC, cNAcC, dNAcC, and vNAcC) and NAc shell subnuclei (NAcLS and NAcMS) using retrograde tracing strategy combined with the interactive WholeBrain framework. We found that NAc neurons integrated inputs from diverse brain regions, from the cerebrum to the brain stem. For the NAcC subdomains, input neurons projecting to the rNAcC and cNAcC showed similar distributions across the same upstream brain regions but from almost non-overlapping populations, whereas those projecting to the dNAcC and vNAcC had relatively different distribution pattern over the same input regions (Figure 9A). The NAc shell subnuclei showed more diverse input patterns from numerous brain areas. The input neurons of NAcMS exhibited a very distinct distribution pattern, mainly concentrating on the HPF, CTXsp, and STR (Figure 9B). The PAL and HY neurons also send innervation to NAcMS but seldom to NAcLS. Input regions of NAcLS and NAcC were similar, but the distributions of input neurons across those regions were different. Both received inputs from a large portion of neurons in the isocortex and OLF. The similarities and differences in their input distribution observed in our study may provide new insights into the diverse functions of the NAc.

Using the WholeBrain framework for brain-wide maps (Fürth et al., 2018), we can quantify the brain-wide distribution of input neurons of different brains and injection sites. We analyzed the input patterns of different subregions of NAcC and shell subdomains in each upstream brain region. Compared with Brog et al. (1993), we found that the NAcMS had distinct upstream brain regions from the NAcC as well as the NAcLS, whereas the NAcC and NAcLS had similar upstream brain regions but a different distribution of input proportions across those regions.

Previous study has also reported differences in the responses to application of the dopamine agonist in rNAcC and cNAcC (Bowers et al., 2000) and to deep brain stimulation of dNAcC and vNAcC (Rodriguez-Romaguera et al., 2012), which can be a result of three types of the input patterns of NAc subregions. First, the rNAcC and cNAcC receive inputs from different upstream brain regions; second, the rNAcC and cNAcC received inputs from the same upstream brain regions but with different input proportions; and, third, the rNAcC and cNAcC receive inputs from different subpopulations of neurons within the same upstream brain regions with same or different input proportions. Our brain-wide NAcC mapping results demonstrated the third one can be a possible explanation.

Previous studies have indicated that dNAcC has the opposite effect on the extinction of fear memory and drug-seeking behaviors. For example, activation of the dNAcC with deep brain stimulation promoted fear memory extinction (Rodriguez-Romaguera et al., 2012) and facilitated drug-seeking behavior (Martínez-Rivera et al., 2016). Our study found that some brain regions, including the ILA and BLA, preferentially innervate to the dNAcC. Earlier research has confirmed the role of the ILA in fear extinction using lesion, drug infusion, and stimulation approaches (Milad and Quirk, 2012), and showed that the BLA→NAc pathway regulated the reinstatement of alcohol-seeking (Baldi and Bucherelli, 2010; Keistler et al., 2017). We found that dNAcC received much more BLA and ILA inputs than vNAcC (Figure 5), indicating that the ILA→dNAcC pathway may be an important circuit involved in fear extinction, whereas the BLA→dNAcC pathway may be involved in the extinction of drug addiction.

We compared the NAcC, as a whole, with the NAcLS and NAcMS in terms of their brain-wide distribution of input neurons. The main upstream regions containing inputs neurons to the NAcC and NAcS were consistent with those reported in a previous retrograde tracking study, which focused on the D1 dopamine receptor (D1R)- and D2R-expressing medium spiny neurons (MSNs) within the NAcC and NAcS (Li et al., 2018). In that research, Li et al. (2018) systematically identified the brain areas projecting to the D1R- and D2R-MSNs in the NAcC and NAcS, whereas we focused on comparing the similarities and differences in upstream brain regions of different subdomains of the NAcC (rNAcC, cNAcC, dNAcC, and vNAcC) and subdomains of the NAcS (NAcLS and NAcMS). Li et al. (2018) found the distributions of input neurons in all upstream brain regions projecting to the NAcC D1R-MSNs and D2R-MSNs were similar, with only 2 out of 84 brain regions showed different proportions of projecting to the NAcC D1R- and D2R-MSNs. We found that the number of input neurons in the same upstream brain regions (9 out of 57) projecting to the dNAcC and vNAcC was slightly different. They found that D1R-MSNs and D2R-MSNs in both NAc subregions receive similar inputs from diverse sources, but we found that NAcLS and NAcMS have different input patterns, and particularly, HY send innervation almost exclusively to NAcMS. Prior functional studies have indicated that the NAcMS received glutamatergic direct inputs from ILA, BLA, and SUB, while received direct GABAergic inputs from ZI (Castro and Bruchas, 2019), which is in accordance with our mapping results. We dissected an exclusive afferent pathway of the NAcMS, which was neither shared with NAcC nor NAcLS. The NAcMS was reported to receive direct inputs from LHA orexin and melanin-concentrating hormone populations, and this connection provided the NAcMS with unique access to metabolic and motivational information (Baldo et al., 2003; Diniz and Bittencourt, 2017). In addition, the caudal nucleus of the solitary tract (NTS) sends long-range catecholamine- and peptide-rich projections directly to NAcMS (Delfs et al., 1998; Wang et al., 2015), this comparatively less studied pathway could directly relay important visceral information to the NAcMS to modulate motivation and stress-related behavior. Altogether, while sharing similarities with the rest of the NAc, the NAcMS possesses a number of unique input sources, indicating that NAcMS might be a more distinct subdomain in both anatomical and functional terms.

Rodents rely heavily on olfactory cues for social interactions; in turn, odor-dependent social learning depends on top-down modulation of the olfactory system (Choe et al., 2015). The AON and PIR are the sensory cortices of olfaction (Linster and Fontanini, 2014). In our study, we found that the AON and PIR accounted for the most projecting to the NAcC and NAcLS, respectively. I can be a straightforward explanation as to why so many studies have found that the NAc plays an important role in social activities in rodents (Dölen et al., 2013; Francis et al., 2015; Walsh et al., 2018). The mPFC is thought to be a center for decision-making, memory (Euston et al., 2012), and social behavior (Ko, 2017). Researchers have proposed that the function of the mPFC is to learn associations between contexts, locations, events, and corresponding adaptive responses, particularly emotional responses (Euston et al., 2012). Our results showed that the mPFC (including the ACA, PL, ILA, and ORB) and other isocortex regions sent innervation preferentially to NAcC and NAcLS respectively. We suspect that the information of learned association from mPFC and sensory/motor information from isocortex may be deployed to different NAc subregions through these different input pathways. However, further experiments are needed to confirm this hypothesis.

The LS is a forebrain structure that receives fibers primarily from the ILA region and moderately from the PL region in rats (Vertes, 2004) and monkeys (Chiba et al., 2001). The LS-mPFC pathway played a role in depression-related behavior (fluoxetine and stress inversely modify LS-mPFC neuronal responsivity), and the CA2-LS pathway is involved in aggressive behavior (Leroy et al., 2018). Previous brain-wide mapping of projections from the LS in mice found a connection between the LS and NAc (Deng et al., 2019), but the input proportion of neurons projecting to the NAc or subnuclei was not quantified. In our study, we found that the LS contributed the greatest input proportion to the NAcMS. However, the function of this LS-NAcMS circuit is still unknown.

The paraventricular nucleus of the TH (PVT) in the TH, is located adjacent to the third VL. PVT neurons encode multiple salient features of sensory stimuli, including reward, aversion, novelty, and surprise (Zhu et al., 2018). It also plays an important role in pain regulation (Chang et al., 2019) and acts as an interface for reward processing to accurately guide reward-seeking behavior (Otis et al., 2019). In addition, PVT glutamatergic neurons control wakefulness through the PVT-NAc pathway (Ren et al., 2018). Interestingly, the PVT is activated by a wide range of stress paradigms (Fernandes et al., 2002), has been found to respond strongly to a wide variety of stressors (Bubser and Deutch, 1999; Heilbronner et al., 2004; Spencer et al., 2004; Heydendael et al., 2012). NAc was relevant to stress susceptibility (Chandra et al., 2017; Heshmati et al., 2018; Muir et al., 2018). The PVT contributed the greatest thalamic input portion to the NAc, which might be important for regulating the alert-stress equilibrium in the brain.

The ENT is divided into the ENTm and ENTl based on their distinctive cytoarchitecture and connectivity patterns. The ENTm contains strongly position-related (spatial) neurons, whereas the ENTl contains neurons encoding object information, attention, and motivation (Yu et al., 2019). In this study, we found that the ENTl and ENTm demonstrated the opposite projecting patterns to the NAcLS, NAcMS, and NAcC. However, the exact role of these different projecting patterns still remains unclear.

CTB retrograde tracing is widely used for elucidating neuronal connectivity; however, it does have several limitations (Köbbert et al., 2000). Although retrograde CTB is useful for marking the identity of cell bodies, as it remains in vesicles and is granular in cells, it cannot provide detailed morphology of neurons. Additionally, although we revealed the input neural circuitries of different NAc subregions, quantification of inputs largely depended on the location of the CTB injection and its diffusion at the injection site. It can be difficult to cover an entire NAc subregion without spilling over to the adjacent areas. To lower the possibility of nonspecific infection, we injected a small volume of CTB and used a very slow injection rate to limit its spread to a small range. We perhaps overlooked some input regions in our experiments because we biased our injections towards smaller volumes and confined regions. In the future, combining new genetic and viral approaches will be necessary to explore the diverse cell subtypes in the NAc with higher specificity.

## Data Availability Statement

The raw data supporting the conclusions of this article will be made available by the authors, without undue reservation, to any qualified researcher.

## Ethics Statement

This study was carried out in accordance with the guidelines issued by the Institutional Animal Care and Use Committee (IACUC) at Huazhong University of Science and Technology, Wuhan, China. All protocols were approved by the IACUC and every effort was made to ensure the mice used were treated humanely and any discomfort was kept to a minimum.

## Author Contributions

LM conceptualized the project, performed most experiments, analyzed the data, and wrote the manuscript with DY. WC conceptualized the project and performed most experiments. DY wrote and edited the manuscript. YH supervised the research, discussion, and writing of the manuscript. All authors read and approved the final manuscript.

## Conflict of Interest

The authors declare that the research was conducted in the absence of any commercial or financial relationships that could be construed as a potential conflict of interest.

## Footnotes

^1^http://wholebrainsoftware.org/

## Abbreviations

aca: Anterior commissure, anterior part
AAA: Anterior amygdalar area
ACA: Anterior cingulate area
AHN: Anterior hypothalamic nucleus
AI: Agranular insular area
AON: Anterior olfactory nucleus
ARH: Arcuate hypothalamic nucleus
ATN: Anterior group of the dorsal thalamus
AUD: Auditory areas
BLA: Basolateral amygdalar nucleus
BMA: Basomedial amygdalar nucleus
BST: Bed nuclei of the stria terminalis
CA1: Hippocampal field CA1
CA2: Hippocampal field CA2
CA3: Hippocampal field CA3
CEA: Central amygdalar nucleus
CLA: Claustrum
CM: Central medial nucleus of the thalamus
COA: Cortical amygdalar area
CP: Caudoputamen
CS: Superior central nucleus raphe
CTXsp: Cortical subplate
DG: Dentate gyrus
DMH: Dorsomedial nucleus of the hypothalamus
DP: Dorsal peduncular area
ECT: Ectorhinal area
ENTl: Lateral entorhinal area
ENTm: Medial entorhinal area
EP: Endopiriform nucleus
EPI: Epithalamus
fx: Columns of the fronixfornix
GU: Gustatory areas
HY: Hypothalamus
HPF: Hippocampal formation
IA: Intercalated amygdalar nucleus
ILA: Infralimbic area
IMD: Intermediodorsal nucleus of the thalamus
LA: Lateral amygdalar nucleus
LHA: Lateral hypothalamic area
LPO: Lateral preoptic area
LS: Lateral septal nucleus
mPFC: Medial prefrontal cortex
MB: Midbrain
MBO: Mammillary body
MD: Mediodorsal nucleus of thalamus
MEA: Medial amygdalar nucleus
MO: Somatomotor areas
MOB: Main olfactory bulb
MPO: Medial preoptic area
MPN: Medial preoptic nucleus
MRN: Midbrain reticular nucleus
MSC: Medial septal complex
NAc: Nucleus accumbens
NAcC: Nucleus accumbens core
rNAcC: Rostral nucleus accumbens core
cNAcC: Caudal nucleus accumbens core
dNAcC: Dorsal nucleus accumbens core
vNAcC: Ventral nucleus accumbens core
NAcS: Nucleus accumbens shell
NAcLS: Lateral nucleus accumbens shell
NAcMS: Medial nucleus accumbens shell
NLOT: Nucleus of the lateral olfactory tract
NST: Solitary tract nucleus
OLF: Olfactory
ORB: Orbital area
OT: Olfactory tubercle
PA: Posterior amygdalar nucleus
PAA: Piriform-amygdalar area
PAG: Periaqueductal gray
PAL: Pallidum
PERI: Perirhinal area
PF: Parafascicular nucleus
PH: Posterior hypothalamic nucleus
PIR: Piriform area
PL: Prelimbic area
PMd: Dorsal premammillary nucleus
PMv: Ventral premammillary nucleus
PT: Parataenial nucleus
PVT: Paraventricular nucleus of the thalamus
RAmb: Midbrain raphe nuclei
RE: Nucleus of reuniens
RH: Rhomboid nucleus
RN: Red nucleus
SI: Substantia innominata
SMT: Submedial nucleus of the thalamus
SS: Somatosensory areas
STR: Striatum
SUB: Subiculum
TEa: Temporal association areas
TH: Thalamus
TR: Postpiriform transition area
TT: Taenia tecta
TU: Tuberal nucleus
VENT: Ventral group of the dorsal thalamus
VL: Lateral ventricle
VMH: Ventromedial hypothalamic nucleus
VISC: Visceral area
VTA: Ventral tegmental area
ZI: Zona incerta.
