# Supplementary material for: Brain-Wide Mapping of Afferent Inputs to Accumbens Nucleus Core Subdomains and Accumbens Nucleus Subnuclei
Source: Front Syst Neurosci. 2020 Mar 18;14:15. doi: 10.3389/fnsys.2020.00015 (PMC7150367; doi:10.3389/fnsys.2020.00015)
Supplement: Supplementary file 3 [file Data_Sheet_1.PDF]

Supplementary Material

Supplementary Figure 1. (A) Schematic injection site in NAcC. (Bi-v) Left, representative coronal brain sections near CTB-488 (green) injection sites in rNAcC; Right, Representative coronal brain sections near CTB-555 (red) injection sites in cNAcC. Scale bar = 400 μm. **Related to figure 1**

Supplementary Figure 2. (A) Schematic injection site in NAcS. (Bi-v) Left, representative coronal brain sections near CTB-488 (green) injection sites in NAcMS; Right, representative coronal brain sections near CTB-555 (red) injection sites in NAcLS. Scale bar = 600 μm. **Related to figure 1**

Supplementary Table 1. Statics for the distributions of input neurons projecting to subregions of NAcC and NAcS. Related to figure 8.

Supplementary Table 2. The number of input cells calculated in all experiments
