# Supplementary figures and images for: Brain-Wide Mapping of Afferent Inputs to Accumbens Nucleus Core Subdomains and Accumbens Nucleus Subnuclei
Source: Front Syst Neurosci. 2020 Mar 18;14:15. doi: 10.3389/fnsys.2020.00015 (PMC7150367; doi:10.3389/fnsys.2020.00015)

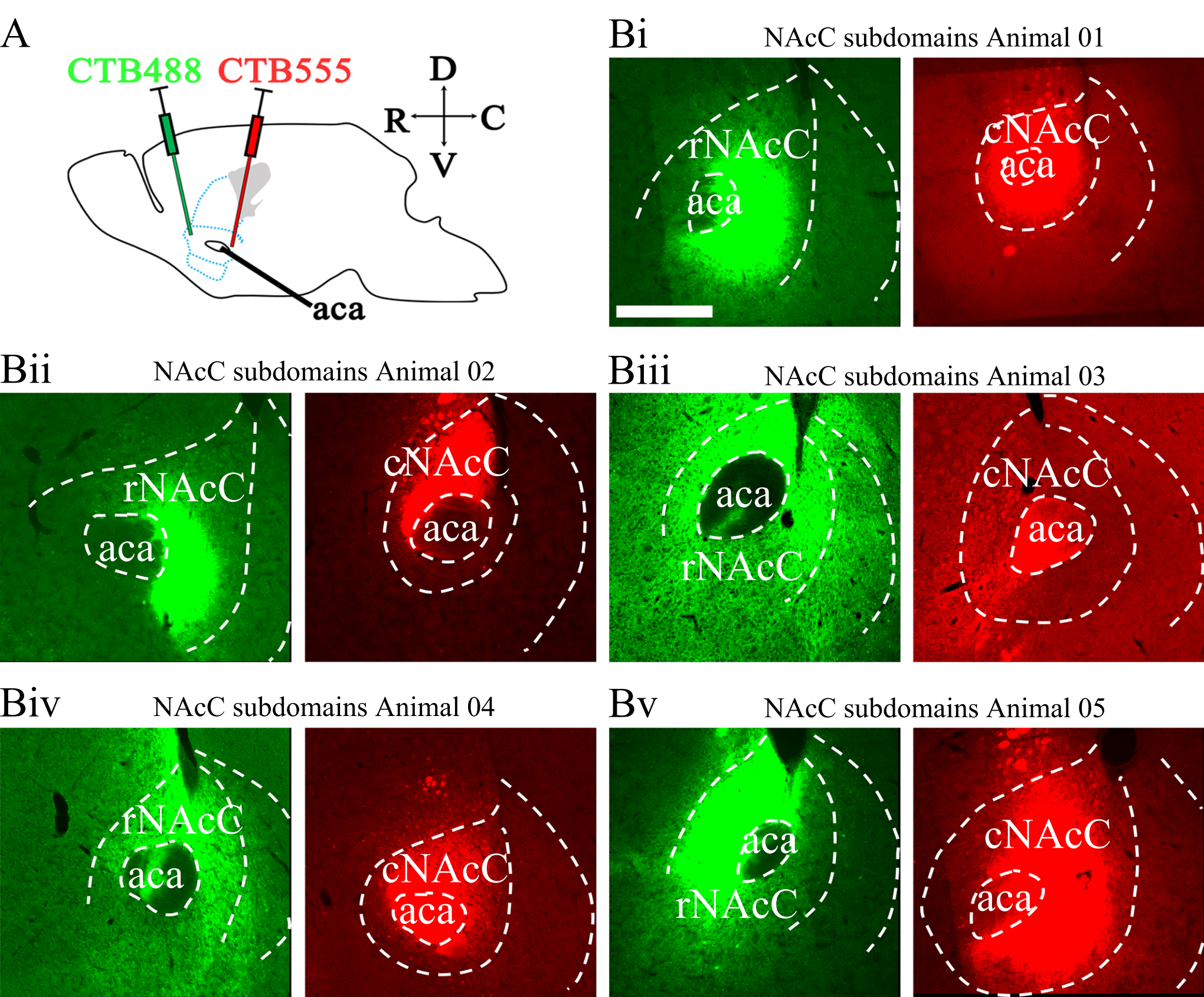

Supplement: Supplementary file 4 [file Image_1.TIF]

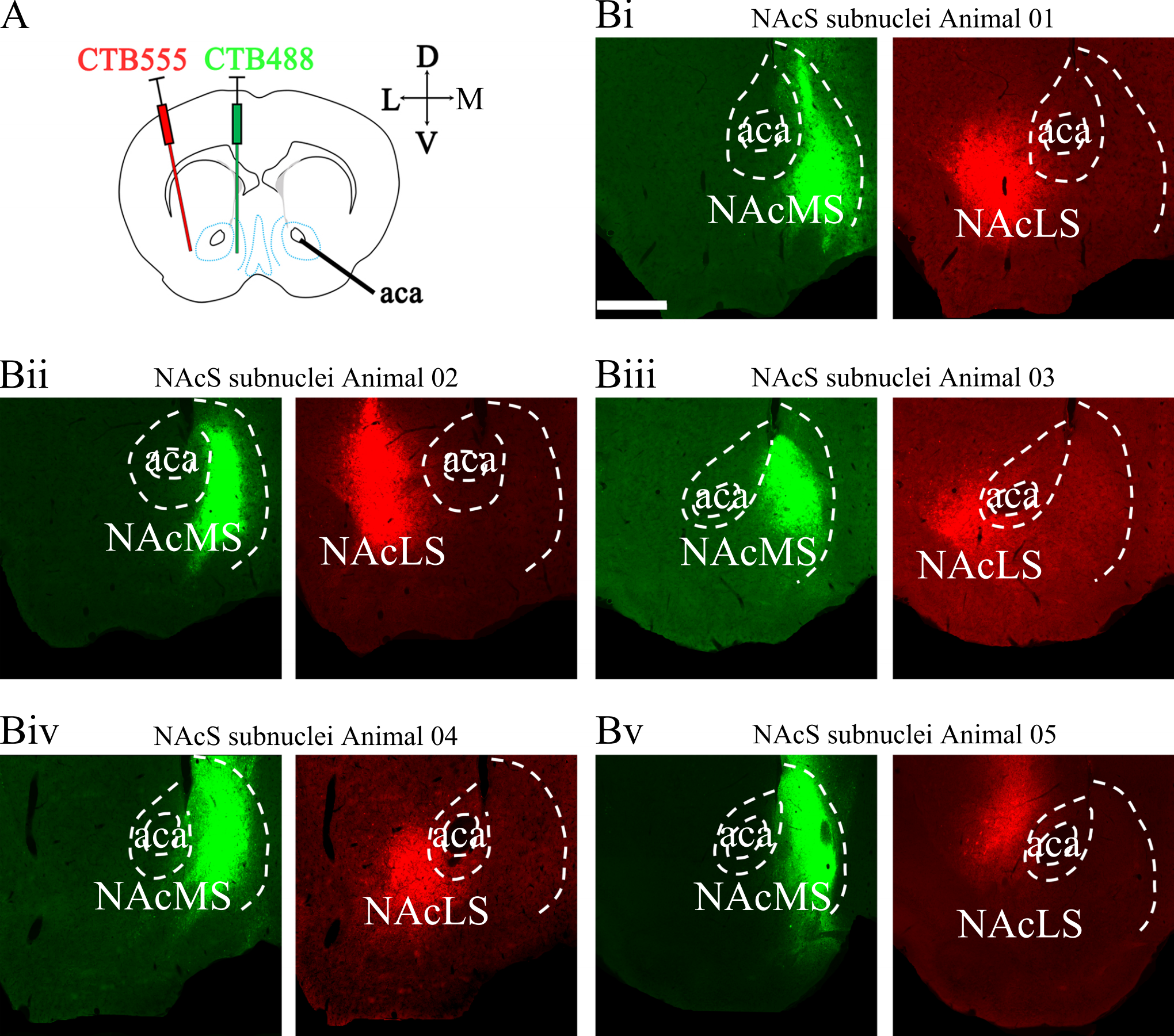

Supplement: Supplementary file 5 [file Image_2.TIF]
